# Antioxidant and Antiadipogenic Activities of Galkeun-Tang, a Traditional Korean Herbal Formula

**DOI:** 10.1155/2014/763494

**Published:** 2014-12-10

**Authors:** Soo-Jin Jeong, Sae-Rom Yoo, Ohn-Soon Kim, Chang-Seob Seo, Hyeun-Kyoo Shin

**Affiliations:** Herbal Medicine Formulation Research Group, Herbal Medicine Research Division, Korea Institute of Oriental Medicine, Daejeon 305-811, Republic of Korea

## Abstract

Galkeun-tang (GKT; *Galgen-tang* in Chinese and *Kakkon-to* in Japanese), a traditional herbal formula, has been used for treatment of the common cold. Here, we report *in vitro* antioxidant and antiadipogenic effects of GKT. GKT increased the activities of scavenging 2,2′-azinobis-(3-ethylbenzothiazoline-6-sulfonic acid) (ABTS) and 2,2-diphenyl-1-picrylhydrazyl (DPPH) radicals. GKT also significantly reduced the malondialdehyde (MDA) generation during low-density lipoprotein (LDL) oxidation and the electrophoretic mobility of oxidized LDL, indicating inhibitory effects of GKT on Cu^2+^-mediated oxidation of LDL. Regarding antiadipogenic activity, GKT treatment significantly suppressed lipid accumulation, triglyceride production, and glycerol-3-phosphate dehydrogenase (GPDH) activity in differentiated 3T3-L1 adipocytes. Consistent with this, GKT significantly reduced the secretion of leptin, a major adipokine, in differentiated 3T3-L1 adipocytes. Overall, our findings suggest that GKT has the potential for antioxidative and antiadipogenic properties.

## 1. Introduction

Oxidative stress is an imbalance between the production of reactive oxygen species (ROS) and antioxidative defenses [1]. Disruption in normal redox signaling can mediate toxic effects and, thus, is closely associated with various human diseases [2]. Obesity is a metabolic disorder caused by excess fat accumulation in adipose tissue [3]. Recent papers have confirmed a critical role for oxidative stress in the pathogenesis of obesity or its associated diseases [4–6]. A change in protection against antioxidative mechanisms was observed in an obese rodent model and in obese human males [7, 8]. Thus, the development of antioxidants could be a valuable approach for treating and preventing obesity or its related diseases. Antioxidative activities of synthetic or natural products have been reported in obesity models. Natural products are considered particularly attractive antiobesity drug candidates because of their higher efficacies and fewer side effects.

Many traditional herbal medicines have attracted increasing attention for their complementary therapeutic effects with few or no side effects compared with Western medicines [9, 10]. Galkeun-tang (GKT;* Galgen-tang* in Chinese and* Kakkon-to* in Japanese), a traditional herbal formula, has been used widely for treatment of the common cold, flu, and fever. In recent research papers, several pharmacological activities of GKT have been reported including antiviral, anti-inflammatory, and immune-regulating activities [11–15]. However, the effect of GKT on obesity or obesity-related diseases has not been reported. The pathogenesis of obesity and its associated diseases is closely related to an inflammatory reaction caused by nutrient excess and imbalance that has been termed “metainflammation” [16]. Thus, we hypothesized that the previously reported anti-inflammatory activity of GKT may influence obesity or its related diseases.

We, therefore, investigated the antioxidant and antiadipogenic effects of GKT. We analyzed its scavenging activities on 2,2′-azinobis-(3-ethylbenzothiazoline-6-sulfonic acid) (ABTS) and 2,2-diphenyl-1-picrylhydrazyl (DPPH) radicals in* in vitro* systems. The effect on low-density lipoprotein (LDL) oxidation was assessed by measuring production of malondialdehyde (MDA). In addition, its inhibitory effects on adipogenesis, the process by which preadipocytes become differentiated adipocytes [17], were determined by Oil Red O staining and assays for triglyceride content, glycerol-3-phosphate dehydrogenase (GPDH) activity, and leptin production in differentiated 3T3-L1 adipocytes.

## 2. Materials and Methods

### 2.1. Plant Materials

The seven herbal medicines forming GKT were purchased from Omniherb (Yeongcheon, Korea) and HMAX (Jecheon, Korea). The origin of these herbal medicines was taxonomically confirmed by Professor Je Hyun Lee, Dongguk University, Gyeongju, Korea. A voucher specimen (2008-KE02-1~KE02-7) has been deposited at the Herbal Medicine Formulation Research Group, Korea Institute of Oriental Medicine.

### 2.2. Preparation of GKT Water Extract

A GKT decoction consisting of seven herbal medicines including Puerariae Radix, Cinnamomi Ramulus, Ephedrae Herba, Paeoniae Radix, Glycyrrhizae Radix et Rhizoma, Zingiberis Rhizoma Crudus, and Zizyphi Fructus was mixed (Table 1; 3.5 kg; 44.0 g × 80) and extracted in a 10-fold mass of water at 100°C for 2 h under pressure (1 kgf/cm^2^) using an electric extractor (COSMOS-660; Kyungseo Machine Co., Incheon, Korea). The water extract was then filtered through a standard sieve (number 270, 53 *μ*m; Chung Gye Sang Gong Sa, Seoul, Korea) and the solution was evaporated to dryness and freeze-dried to give a powder. The yield of GKT water extract was 12.6% (441.6 g).

### 2.3. ABTS Radical Scavenging Activity

The free radical scavenging activity of GKT extract on ABTS was assessed by the method described by Re et al. [18] with slight modifications. ABTS radical cation was produced by reacting 7 mM ABTS solution with 2.45 mM potassium persulfate in the dark at room temperature for 16 h. Absorbance of the reactant was later adjusted to 0.7 at a wavelength of 734 nm. A 100 *μ*L aliquot of GKT solution at different concentrations (12.5–200 *μ*g/mL) was mixed with 100 *μ*L ABTS^•+^ solution. The reaction mixture was incubated for 5 min in the dark at room temperature. The absorbance of the resulting solution was measured at 734 nm with a spectrophotometer (Benchmark Plus, Bio-Rad, Hercules, CA). The radical scavenging capacity of the tested samples was calculated using the following equation:

(1) Scavenging  activity%=1−Absorbance  of  sampleAbsorbance  of  control×100.

### 2.4. DPPH Radical Scavenging Activity

The free radical scavenging activity of GKT extract on DPPH was assessed using the method described by Moreno et al. [19]. In brief, a 100 *μ*L aliquot of GKT sample solution at different concentrations (50–400 *μ*g/mL) was mixed with 100 *μ*L DPPH solution (0.15 mM in methanol). The reaction mixture was incubated for 30 min in the dark at room temperature. The absorbance of the resulting solution was measured at 517 nm with a spectrophotometer (Benchmark Plus, Bio-Rad, Hercules, CA). The radical scavenging capacity of the tested samples was calculated using the above formula.

### 2.5. Oxidation of LDL by CuSO_4_

LDL samples (500 *μ*g protein/mL, Biomedical Technologies, Stoughton, MA) were prepared at 37°C in a medium containing 10 mM phosphate buffer (pH 7.4) and various concentrations (15.7–250 *μ*g/mL) of GKT extract. After 5 min, oxidation was initiated by the addition of CuSO_4_ (25 *μ*M). After 6 h of oxidation, lipid peroxidation (Section 2.6) and electrophoretic mobility (Section 2.7) of the LDL were measured as described below.

### 2.6. Determination of MDA Using TBARS Assay

Lipid peroxidation of LDL was estimated by the determination of the level of MDA using a TBARS assay kit (Bioassay Systems LLC, Hayward, CA) according to the manufacturer's protocols. After oxidation, 50 *μ*g of LDLs was mixed with 200 *μ*L of thiobarbituric acid (TBA) and incubated at 100°C for 30 min. After completing the reaction, the absorbance at 535 nm was measured using a spectrophotometer.

### 2.7. Relative Electrophoretic Mobility (REM) Assay

The electrophoretic mobility of LDLs was measured using agarose gel (0.8% agarose in TAE buffer) electrophoresis and Coomassie Brilliant Blue R-250 staining. Electrophoresis was performed at 100 V for 30 min. REM was defined as the ratio of the distances migrated from the origin by oxidized LDL (oxLDL) versus native LDL.

### 2.8. Cell Culture and Differentiation

Mouse preadipocyte cell line 3T3-L1 was obtained from the American Type Culture Collection (ATCC, CL-173, Rockville, MD). The cells were cultured in DMEM (Gibco BRL, Carlsbad, CA) supplemented with 10% newborn calf serum (NCS, Gibco BRL, Carlsbad, CA) at 37°C. For adipocyte differentiation, the cells were stimulated with 3T3-L1 differentiation medium containing isobutylmethylxanthine, dexamethasone, and insulin (MDI) (Zenbio Inc., Research Triangle Park, NC) for 48 h after reaching confluence. The medium was switched to DMEM containing 10% fetal bovine serum (FBS) and 1 *μ*g/mL insulin after 2 days and then changed to DMEM containing 10% FBS for an additional 4 days. Cells were treated with GKT extract (0, 25, 50, 100, 200, or 400 *μ*g/mL) during 8 days of the differentiation period. GW9662 (Sigma, St. Louis, MO), a peroxisome proliferator-activated receptor-gamma (PPAR-*γ*) antagonist, was used as a positive control.

### 2.9. Cytotoxicity Assay

Undifferentiated 3T3-L1 cells were treated with various concentrations of GKT for 24 h. To obtain differentiated adipocytes, 3T3-L1 preadipocytes were differentiated for 8 days in the presence of GKT. The cells were incubated with GKT extract at 37°C under 5% CO_2_. Cell counting kit- (CCK-) 8 solution (Dojindo, Kumamoto, Japan) was added and incubated for 4 h. After incubation, the absorbance was read at 450 nm using a microplate reader (Benchmark Plus, Bio-Rad, Hercules, CA).

### 2.10. Oil Red O Staining

3T3-L1 preadipocytes were differentiated into adipocytes by adding isobutylmethylxanthine, dexamethasone, and insulin (MDI) for 8 days. The cells were treated with or without GKT (200 *μ*g/mL) or GW9662 (20 *μ*M) during the differentiation period. The differentiated 3T3-L1 cells were fixed with 10% formalin for 15 min at room temperature and washed with 70% ethanol and phosphate-buffered saline (PBS). The cells were stained with Oil Red O (Sigma, St. Louis, MO) for 5 min and then washed with PBS. Cell images were collected using an Olympus CKX41 inverted microscope (Olympus, Tokyo, Japan). Stained oil droplets were dissolved in isopropyl alcohol and measured by reading the absorbance at 520 nm.

### 2.11. Triglyceride Quantification Assay

3T3-L1 preadipocytes were differentiated into adipocytes by adding MDI for 8 days. The cells were treated with or without GKT (25, 50, 100, 200, or 400 *μ*g/mL) or GW9662 (20 *μ*M) during the differentiation period. The triglyceride content of cells was enzymatically measured using a commercial kit (BioVision Inc., Milpitas, CA). Briefly, the 3T3-L1 adipocytes treated with GKT were homogenized in 5% NP-40 assay buffer and the sample was slowly heated to solubilize all triglycerides. The samples were mixed with lipase and triglyceride reaction mixture. After 1 h of incubation, the sample absorbance was measured at 570 nm.

### 2.12. GPDH Activity Assay

After the induction of adipocyte differentiation in the presence of GKT, 3T3-L1 cells were washed twice with PBS. GPDH activity was determined by using a commercial kit (TAKARA, Tokyo, Japan) and monitoring the dihydroxyacetone phosphate-dependent oxidation of nicotinamide adenine dinucleotide (NADH) at 340 nm and the results were expressed as unit/mg of protein.

### 2.13. Leptin Immunoassay

Leptin levels were assayed using a mouse leptin immunoassay kit (R&D Systems, Minneapolis, MN) according to the manufacturer's instructions. In brief, the culture supernatant was collected from the differentiated 3T3-L1 with or without GKT treatment. An equal ratio of the supernatants (50 *μ*L) and assay diluent RD1W (50 *μ*L) was added to wells of a 96-well plate and incubated for 2 h at room temperature. After washing 5 times with 400 *μ*L of wash buffer, 100 *μ*L of mouse leptin conjugate was added to each well and incubated for 2 h at room temperature. After washing 5 times, 100 *μ*L of substrate solution was added to each well and incubated for 30 min at room temperature in the dark. Finally, 100 *μ*L of stop solution was added to each well and the absorbance was measured at 450 nm.

### 2.14. Chemicals and Reagents for HPLC Analysis

Cinnamaldehyde, cinnamic acid, glycyrrhizin, liquiritin, paeoniflorin, and puerarin (all purity ≥ 98.0%) were purchased from Wako Fine Chemicals (Osaka, Japan). The HPLC-grade reagents methanol, acetonitrile, and water were obtained from J. T. Baker (Phillipsburg, NJ). Glacial acetic acid was obtained from Junsei Chemical Co. (Tokyo, Japan).

### 2.15. Preparation of Standard and Sample Solutions for HPLC Analysis

Methanol standard stock solutions were made containing each of cinnamic acid, glycyrrhizin, liquiritin, paeoniflorin, and puerarin at 1.0 mg/mL. Cinnamaldehyde was dissolved in methanol at a concentration of 1.05 mg/mL. The stock solutions were stored below 4°C.

For HPLC analysis, lyophilized GKT extract was weighed (200 mg) into a 20 mL flask and distilled water was added to the volumetric mark, and then the mixture was passed through a 0.2 *μ*m syringe filter before injection into the HPLC system.

### 2.16. HPLC Analysis of GKT

HPLC was performed on a Shimadzu LC-20A HPLC system (Shimadzu Co., Kyoto, Japan) consisting of a solvent delivery unit, an on-line degasser, a column oven, an autosampler, and a photodiode array (PDA) detector. The data processor employed LCsolution software (Version 1.24). The analytical column used was a Gemini C_18_ (250 × 4.6 mm; particle size 5 *μ*m, Phenomenex, Torrance, CA) maintained at 40°C. The mobile consisted of solvent A (1.0%, v/v, acetic acid in H_2_O) and solvent B (1.0%, v/v, acetic acid in acetonitrile). The gradient flow was as follows: 5–70% B for 0–40 min, 70–100% B for 40–45 min, 100% B for 45–50 min, and 100–5% B for 55 min. The flow rate was 1.0 mL/min and the injection volume was 10 *μ*L. The quantitative analysis of the six compounds was carried out at 230 nm (paeoniflorin), 254 nm (glycyrrhizin and puerarin), and 280 nm (cinnamaldehyde, cinnamic acid, and liquiritin).

### 2.17. Statistical Analysis

All data were presented as mean ± standard error of the mean (S.E.M.). Group differences were assessed by one-way ANOVA and post hoc Tukey's multiple comparison test using Graphpad InStat ver.3.10 (Graphpad Software, Inc., San Diego, CA). Significance of differences from the normal control was taken as *P* < 0.05.

## 3. Results

### 3.1. HPLC Analysis of GKT

**Figure 1:**
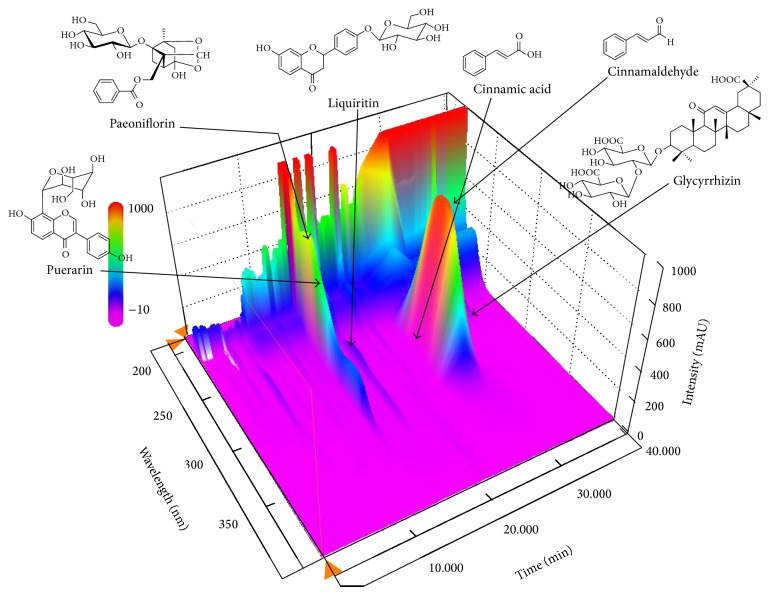
Three-dimensional chromatogram of GKT by HPLC-PDA.

All calibration curves were obtained by assessment of peak areas from standard solutions in the concentration ranges: puerarin, cinnamic acid, and glycyrrhizin, 2.34–300.00 *μ*g/mL; paeoniflorin, liquiritin, and cinnamaldehyde, 0.78–100.00 *μ*g/mL, 1.56–200.00 *μ*g/mL, and 1.64–105.00 *μ*g/mL, respectively. The retention times were 13.81, 15.72, 17.65, 25.77, 28.08, and 33.55 min for puerarin, paeoniflorin, liquiritin, cinnamic acid, cinnamaldehyde, and glycyrrhizin, respectively. The calibration curves, correlation coefficients (*R* ^2^), limit of detection (LOD), and limit of quantification (LOQ) of the six marker compounds are summarized in Table 2. Using optimized chromatography conditions, a three-dimensional chromatogram was obtained using the HPLC-PDA detector (Figure 1). The concentrations of the six marker compounds were 2.01–12.17 mg/g and are summarized in Table 3.

### 3.2. Antioxidant Activity of GKT

To evaluate the antioxidant activity of GKT, we tested its scavenging activities on ABTS and DPPH radicals. The ABTS radical scavenging activity of GKT is presented in Table 4. The extracts of GKT showed a dose-dependent radical scavenging activity. The concentration of GKT required for 50% inhibition (IC_50_) of ABTS radicals was 51.16 *μ*g/mL, while the IC_50_ value of ascorbic acid, as a positive control, was 3.22 *μ*g/mL. The antioxidant activities obtained for GKT by the DPPH method are shown in Table 5. Similar to the ABTS assay, GKT reduced DPPH radical formation in a concentration-dependent manner. The IC_50_ of GKT against DPPH radicals was 242.96 *μ*g/mL, while the IC_50_ value of ascorbic acid was 10.43 *μ*g/mL.

### 3.3. Effect of GKT on Cu^2+^-Mediated Oxidation of LDL

**Figure 2:**
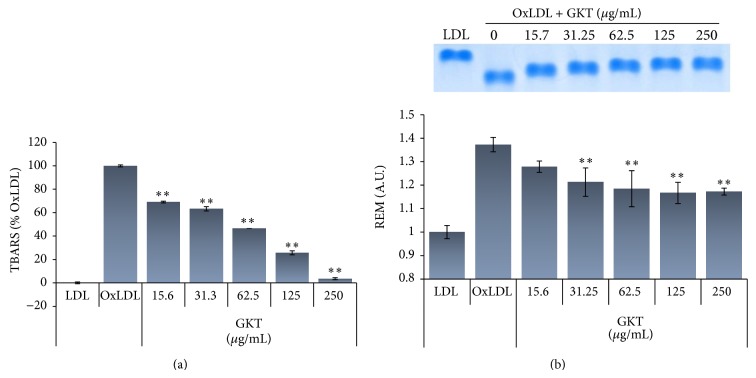
Inhibitory effects of GKT on Cu^2+^-induced LDL oxidation. The indicated concentrations of GKT and LDLs were incubated with CuSO_4 _for 6 h at 37°C. The TBARS level (a) and electrophoretic mobility (b) of LDLs were measured using a TBARS assay kit and agarose gel electrophoresis, respectively. The data are presented as the mean values of three experiments' ± S.E.M. ^**^ *P* < 0.01 compared with the oxLDL group.

The generation of MDA equivalents during LDL oxidation was estimated by the TBARS assay. As shown in Figure 2(a), when LDL was incubated with CuSO_4_ for 6 h, a significant increase in TBARS was detected. In contrast, GKT significantly reduced the amount of TBARS formed in a dose-dependent manner (IC_50_: 53.23 *μ*g/mL). The alteration of mobility in agarose gel electrophoresis reflects the increase in the negative charge of LDL particles which occurs during oxidation [20]. When the oxidation was carried out in the presence of GKT, the increase in electrophoretic mobility of oxLDL was significantly reduced (Figure 2(b)). These data suggest that GKT has an inhibitory effect on LDL oxidation.

### 3.4. Cytotoxic Effects of GKT in 3T3-L1 Cells

**Figure 3:**
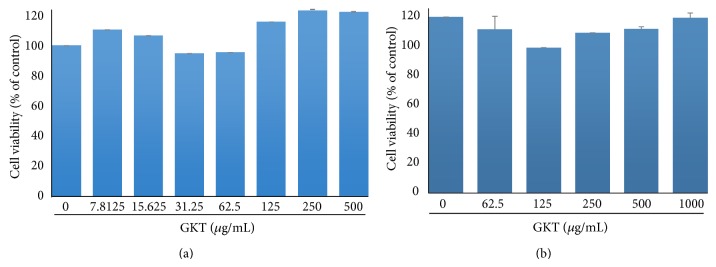
Cytotoxic effects of GKT extract in 3T3-L1 cells. (a) Undifferentiated 3T3-L1 cells were treated with various concentrations of GKT (0, 7.8125, 15.625, 31.25, 62.5, 125, 250, or 500 *μ*g/mL) for 24 h. (b) Adipocyte differentiation was induced by adding isobutylmethylxanthine, dexamethasone, and insulin (MDI) to 3T3-L1 preadipocytes for 8 days. The cells were exposed to various concentrations of GKT (0, 62.5, 125, 250, 500, or 1000 *μ*g/mL) during the differentiation period. Cell viability was determined using a CCK-8 assay kit by measuring the absorbance at 450 nm. Data are presented as the mean ± S.E.M.

To evaluate the possible cytotoxicity of GKT against 3T3-L1 preadipocytes, the cells were treated with various concentrations of GKT for 24 h. As shown in Figure 3(a), GKT had no cytotoxicity against 3T3-L1 preadipocytes. Additionally, the cytotoxicity of GKT was assessed in the differentiated adipocytes. During differentiation for 8 days, the cells were exposed to various concentrations of GKT. No significant cytotoxic effect was observed in GKT-treated adipocytes (Figure 3(b)).

### 3.5. The Inhibitory Effects of GKT on Adipogenesis of 3T3-L1 Adipocytes

**Figure 4:**
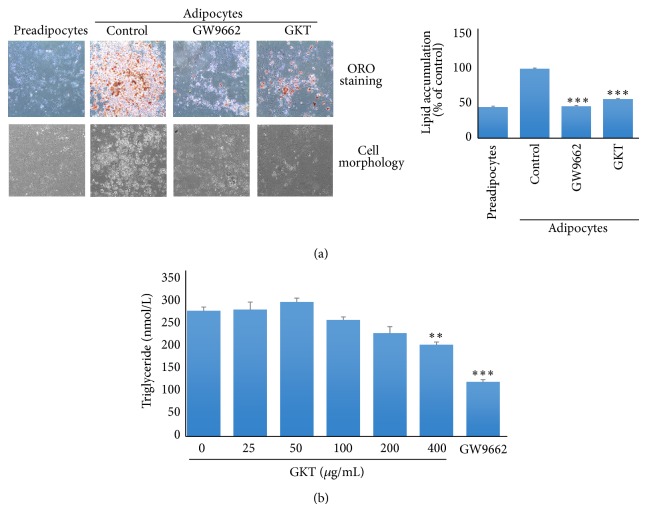
Inhibitory effect of GKT extract on triglyceride accumulation in 3T3-L1 adipocytes. 3T3-L1 preadipocytes were differentiated into adipocytes by adding isobutylmethylxanthine, dexamethasone, and insulin (MDI) for 8 days. The cells were treated with or without GKT or GW9662 (20 *μ*M) during the differentiation period. (a and b) Lipid accumulation in the cells was analyzed by Oil Red O staining. (a) The cells stained with Oil Red O were visualized using an Olympus CKX41 inverted microscope at ×200 magnification (left panel). Stained oil droplets were dissolved in isopropyl alcohol and measured by reading absorbance at 520 nm (right panel). (b) The content of triglyceride was enzymatically measured at 570 nm using a commercial kit (BioVision Inc., Milpitas, CA). Data are presented as the mean ± S.E.M. ^**^ *P* < 0.01 and ^***^ *P* < 0.001 compared with the differentiated control.

Triglyceride production is one of the important events which occurs during adipogenesis [21]. Oil Red O staining was carried out to examine lipid accumulation in the differentiated 3T3-L1 adipocytes. The number of lipid droplets detectable with Oil Red O staining was obviously increased in adipocytes compared with preadipocytes (Figure 4(a)). However, GKT treatment significantly reduced lipid accumulation compared with the untreated differentiated cells. In addition, the intracellular triglyceride content was measured in 3T3-L1 adipocytes treated with GKT. Consistent with the results of Oil Red O staining, triglyceride production was significantly increased in the differentiated adipocytes compared with preadipocytes, but GKT significantly inhibited triglyceride production compared with untreated differentiated cells (Figure 4(b)).

**Figure 5:**
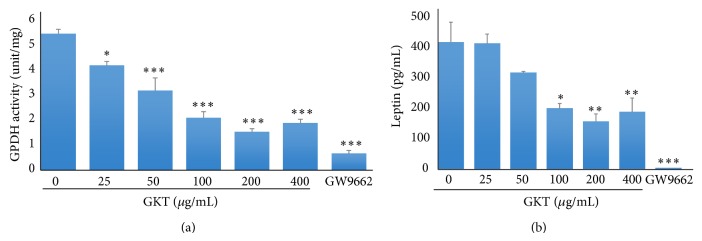
Inhibitory effects of GKT on adipogenesis-related factors in 3T3-L1 adipocytes. 3T3-L1 preadipocytes were differentiated into adipocytes by adding isobutylmethylxanthine, dexamethasone, and insulin (MDI) for 8 days. The cells were exposed to various concentrations of GKT during the differentiation period. (a) GPDH activity of the cells was assessed by measuring the decrease in NADH at 340 nm using the TAKARA GPDH activity assay kit. (b) Culture supernatant was collected from the GKT-treated cells. Leptin production was determined by ELISA at 540 nm subtracted from 450 nm using a mouse leptin immunoassay kit (R&D Systems). Data are presented as the mean ± S.E.M. ^***^ *P* < 0.001, ^**^ *P* < 0.01, and ^*^ *P* < 0.05 compared with the differentiated control.

GPDH is an enzyme that generates glycerol-3-phosphate from dihydroxyacetone phosphate for lipid biosynthesis in adipocytes [22]. As shown in Figure 5(a), treatment with GKT at the concentration of 25~400 *μ*g/mL significantly inhibited the activity of GPDH compared with untreated differentiated adipocytes. Consistent with this, GKT also had a suppressive effect on secretion of leptin, a key adipokine [23], compared with the untreated differentiated cells (Figure 5(b)). Similarly, treatment with GW9662, the positive control, dramatically inhibited adipogenesis in 3T3-L1 cells.

## 4. Discussion

In the present study, we demonstrate that a traditional herbal medicine GKT has antioxidant and antiadipogenesis properties. For quality control of the GKT extract, we performed a quantitative determination of the six main components in GKT using HPLC coupled with a PDA. The investigated components were as follows: puerarin form Puerariae Radix, cinnamaldehyde and cinnamic acid from Cinnamomi Ramulus, paeoniflorin from Paeoniae Radix, and liquiritin and glycyrrhizin from Glycyrrhizae Radix et Rhizoma. The optimized HPLC-PDA method was applied for simultaneous quantitation of the six components in GKT (Figure 1). Among these components, puerarin and glycyrrhizin, which are marker components of Puerariae Radix and Glycyrrhizae Radix et Rhizoma, were detected at 10.29 mg/g and 12.17 mg/g, respectively, as the major components of GKT (Table 3). The establishment of this HPLC-PDA method will be helpful in improving quality control of GKT.

In* in vitro *assay systems, GKT showed significant scavenging effects against ABTS and DPPH radicals (Table 4), and it inhibited LDL oxidation mediated by CuSO_4_ in a dose-dependent manner (Figure 2). In addition, GKT revealed antiadipogenic activity by blocking lipid accumulation, triglyceride production, GPDH activity, and leptin production in adipocytes without cytotoxic effects (Figures 3–5).

Oxidative stress plays an important role in the pathogenesis of various diseases including obesity. Thus, the “oxidative stress paradigm” is an appealing concept for developing novel therapeutics [24]. Excess oxidative stress in obese patients coincides with fat accumulation in adipocytes [4]. We induced the cellular differentiation of 3T3-L1 mouse preadipocytes into adipocytes. This cell line is useful for studying adipogenesis [25]. After adipocyte differentiation, a significant increase of intracellular lipid accumulation compared with untreated preadipocytes was observed by Oil Red O staining. In contrast, GKT significantly inhibited the amount of lipid accumulation compared with untreated differentiated adipocytes (Figure 4(a)). Microscopy further confirmed the inhibitory effect of GKT on adipogenesis. GKT treatment markedly reduced the increase in the number and size of adipocytes, a hallmark of adipocyte endocrine function [26]. Lipid droplets consist of a core of lipid esters and a surface lined with a phospholipid monolayer, and, in adipocytes, the lipid ester core contains triglycerides [27–29]. Consistent with the results of Oil Red O staining, GKT significantly decreased the content of triglyceride in adipocytes (Figure 4(b)). Furthermore, we evaluated the GPDH activity in differentiated 3T3-L1 adipocytes. GPDH is activated strongly in mature adipocytes and plays a role in the triglyceride biosynthesis pathway [30, 31]. GPDH enzyme activity was significantly decreased in GKT-treated adipocytes (Figure 5(a)). The level of another key factor in adipogenesis, leptin, was measured in adipocytes differentiated in the absence or presence of GKT. Leptin is an adipokine exclusively produced by adipocytes in proportion to triglyceride accumulation [32]. As expected, GKT significantly inhibited the level of secretion of leptin in differentiated 3T3-L1 adipocytes (Figure 5(b)). Similar to our study, several groups have reported dual effects of natural products, including purple sweet potato [33], safflower seed [34], and buckwheat sprout [35], on oxidative stress and adipogenesis. Together, these results suggest the potential of natural products, including herbal medicines, as adipogenesis-preventing substances with antioxidant properties.

Interestingly, several herbal components of GKT have been reported to have antioxidant properties or antiobesity effects.* Pueraria lobata *extract ameliorated impaired glucose and lipid metabolism in obese mice [36].* Pueraria lobata *extract also inhibited* tert*-butyl hydroperoxide-induced ROS generation [37]. An extract of* Cinnamomum cassia* twigs inhibited adipocyte differentiation via activation of the insulin signaling pathway in 3T3-L1 preadipocytes [38].* Ephedra sinica *extract exerted antioxidant effects by accelerating free radical scavenging activity and oxidant reducing power [39]. These data could support antiadipogenic/antioxidant activities of GKT. Additional studies will be required to confirm the antioxidant/antiobesity effects of GKT using a high fat diet-fed obese animal model.

## Figures and Tables

**Table 1:** Composition of GKT.

Herbal medicine	Scientific name	Supplier	Source	Amount (g)
Puerariae Radix	*Pueraria lobata *	Omniherb	Jecheon, Korea	9.0
Cinnamomi Ramulus	*Cinnamomi cassia *	HMAX	China	6.0
Ephedrae Herba	*Ephedra sinica *	HMAX	China	6.0
Paeoniae Radix	*Paeonia lactiflora *	Omniherb	Hwasun, Korea	6.0
Glycyrrhizae Radix et Rhizoma	*Glycyrrhiza uralensis *	HMAX	China	6.0
Zingiberis Rhizoma Crudus	*Zingiber officinale *	Omniherb	Yeongcheon, Korea	6.0
Zizyphi Fructus	*Ziziphus jujuba *	Omniherb	Yeongcheon, Korea	5.0

Total amount				44.0

**Table 2:** Regression data, linear range, correlation coefficient, LOD, and LOQ for marker compounds (*n* = 3).

Compound	Linear range (*μ*g/mL)	Regression equation^a^	Correlation coefficient (*R* ^2^)	LOD^b^ (*μ*g/mL)	LOQ^c^ (*μ*g/mL)
Puerarin	2.34–300.00	*y* = 39814.06*x* + 35976.26	1.0000	0.04	0.15
Paeoniflorin	0.78–100.00	*y* = 38738.30*x* − 15273.19	0.9999	0.05	0.15
Liquiritin	1.56–200.00	*y* = 18495.04*x* + 12062.72	1.0000	0.03	0.11
Cinnamic acid	2.34–300.00	*y* = 27338.38*x* + 31461.88	1.0000	0.02	0.06
Cinnamaldehyde	1.64–105.00	*y* = 122000.31*x* + 62587.19	1.0000	0.01	0.03
Glycyrrhizin	2.34–300.00	*y* = 8266.43*x* + 5843.51	1.0000	0.36	1.21

^a^ *y*: peak area (mAU) of compounds; *x*: concentration (*μ*g/mL) of compounds.

^b^LOD = 3 × signal-to-noise ratio.

^c^LOQ = 10 × signal-to-noise ratio.

**Table 3:** Contents of six compounds in the GKT by HPLC (*n* = 3).

Compound	Mean (mg/g)	SD	RSD (%)	Source
Puerarin	10.29	0.02	0.21	Puerariae Radix
Paeoniflorin	3.55	0.02	0.69	Paeoniae Radix
Liquiritin	6.64	0.05	0.75	Glycyrrhizae Radix et Rhizoma
Cinnamic acid	2.01	0.05	2.44	Cinnamomi Ramulus
Cinnamaldehyde	7.30	0.04	0.50	Cinnamomi Ramulus
Glycyrrhizin	12.17	0.09	0.76	Glycyrrhizae Radix et Rhizoma

**Table 4:** Scavenging effects of GKT on ABTS^•+^.

Sample	Concentration (*μ*g/mL)	Scavenging effect (%)	IC_50_^1^ (*μ*g/mL)
GKT^2^	12.5	19.24 ± 1.19	51.16 ± 2.67
	25	32.85 ± 2.80	
	50	58.08 ± 3.51	
	100	82.35 ± 0.26	
	200	97.46 ± 0.30	

AA^3^	1.25	21.15 ± 0.19	3.22 ± 0.06
	2.5	40.61 ± 1.44	
	5	75.86 ± 1.06	

^1^Concentration required for 50% reduction of ABTS^•+^ at 5 min reaction.

^2^Galkeun-tang and ^3^ascorbic acid.

Each value is the mean ± S.E.M. of triplicate determinations.

**Table 5:** Scavenging effects of GKT on DPPH.

Sample	Concentration (*μ*g/mL)	Scavenging effect (%)	IC_50_^1^ (*μ*g/mL)
GKT^2^	50	6.33 ± 0.14	242.96 ± 2.66
	100	22.64 ± 0.49	
	200	47.66 ± 1.07	
	400	78.70 ± 0.60	

AA^3^	1.25	21.15 ± 0.19	3.22 ± 0.06
	2.5	40.61 ± 1.44	
	5	75.86 ± 1.06	

^1^Concentration required for 50% reduction of DPPH at 30 min reaction.

^2^Galgeun-tang and ^3^ascorbic acid.

Each value is the mean ± S.E.M. of triplicate determinations.
